# Sustained Control of Hyperthyroidism in Graves’ Disease: Comparison of Thyroidectomy and Long-Term Antithyroid Treatment

**DOI:** 10.5812/ijem-164940

**Published:** 2026-01-31

**Authors:** Hengameh Abdi, Miralireza Takyar, Ladan Mehran, Safdar Masoumi, Atieh Amouzegar, Fereidoun Azizi

**Affiliations:** 1 Endocrine Research Center, Research Institute for Endocrine Disorders, Research Institute for Endocrine Sciences, Shahid Beheshti University of Medical Sciences, Tehran, Iran

**Keywords:** Graves’ Disease, Hyperthyroidism, Thyroidectomy, Methimazole

## Abstract

**Background:**

The achievement of sustained euthyroidism in patients with Graves’ hyperthyroidism may reduce the risk of mortality and cardiovascular events.

**Objectives:**

We compared the time to euthyroidism and the time remaining in euthyroidism following total thyroidectomy and long-term methimazole (LT-MMI) treatment.

**Methods:**

In this prospective cohort study, two hundred and eight patients with diffuse toxic goiter, 104 (84 women) with total thyroidectomy, and 104 (84 women) on LT-MMI were compared. All patients received adequate levothyroxine treatment after thyroidectomy. Data on serum free thyroxine (fT4), triiodothyronine (T3), and thyrotropin (TSH) every 6 months until the end of follow-up were analyzed. The time to normalization of serum thyroid hormones and TSH concentrations and the percentage of time that thyroid hormones and TSH levels remained within the normal range during a mean follow-up of 11 years were compared between the two study groups using t-test, Mann-Whitney U, chi-square, and Fisher exact tests.

**Results:**

The mean time to euthyroidism was 4.74 ± 2.42 and 4.48 ± 2.54 months in the thyroidectomy and LT-MMI groups, respectively. During follow-up, the percentage of time spent in euthyroidism was 82.67 ± 7.33% vs 94.21 ± 6.98% in the thyroidectomy and LT-MMI groups, respectively (P < 0.001). Patients with thyroidectomy spent more time in subclinical and clinical hypo- and hyperthyroidism compared to LT-MMI patients. Permanent hypocalcemia and vocal cord paralysis occurred in 2 and 1 patients, respectively.

**Conclusions:**

Treatment of hypothyroidism after total thyroidectomy was accompanied by less sustained euthyroidism during long-term follow-up compared with LT-MMI therapy. This difference may be important for the risk of mortality and cardiovascular events, demanding long-term studies for comparison of these outcomes in patients treated with total thyroidectomy and LT-MMI treatment.

## 1. Background

Diffuse toxic goiter or Graves’ disease, caused by autoimmune dysregulation, is the most common cause of hyperthyroidism and may be associated with increased risk of cardiovascular, skeletal, and neuropsychiatric abnormalities and impaired quality of life (1-4). Treatment of hyperthyroidism has always been a dilemma. It has evolved over the last few decades with the introduction of antithyroid drug (ATD) therapy and radioactive iodine (RAI) administration in the 1940s (1, 5). None of the three therapeutic modalities can establish lifelong euthyroidism in patients with Graves’ disease. Approximately 50% of patients experience a relapse of hyperthyroidism after 12 - 24 months of conventional ATD treatment (6). Lifelong hypothyroidism after ablation with RAI therapy or surgery has been the primary concern of patients with Graves’ disease, and both patients and physicians prefer ATD treatment (7). Methimazole (MMI) is the drug of choice for the initial management of hyperthyroidism (8, 9). Recent studies have emphasized that low serum thyrotropin (TSH) concentrations may significantly increase the risk of mortality and cardiovascular events, which correlates with the cumulative period of low-TSH status (10-14). This finding prompts physicians to carefully select treatment modalities to resolve TSH suppression rapidly and to offer more sustained euthyroidism during years of follow-up. It has been shown that surgery as an initial treatment has a lower chance of all-cause mortality, cardiovascular diseases, atrial fibrillation, diabetes, and hypertension compared to RAI or conventional ATD treatment (15). A recent study has reported lower long-term risks of major adverse cardiovascular events (MACE) in patients undergoing surgery compared with patients receiving ATD; in addition, RAI also had a lower MACE risk than conventional ATD therapy (16).

We have demonstrated that long-term ATD (LT-ATD) treatment is effective in achieving euthyroidism in patients with Graves’ disease for years and overcomes the increased relapse rate of conventional ATD treatment by decreasing the rate to 15% during 4 years of ATD withdrawal (17-19). Others have proposed this mode of treatment because of the low relapse rate and infrequent adverse events after the first year of ATD treatment (20-22). It has been shown that LT-ATD therapy is associated with a shorter “time to euthyroidism” and longer “time remained in euthyroidism” compared to RAI treatment (19). This finding may postulate a favorable role of long-term methimazole (LT-MMI) for less frequent cardiovascular events.

## 2. Objectives

Since there is no report of such a comparison between LT-MMI and thyroidectomy, the present study aimed to compare the time of achievement of euthyroidism and the sustainability of normal serum TSH during long-term follow-up of patients treated with LT-ATD therapy and thyroidectomy.

## 3. Methods

Patients from the towards outstanding hyperthyroid care induced by antithyroid drugs (TOHID) cohort were selected for this study (19). Towards outstanding hyperthyroid care induced by antithyroid drugs trials have been registered in the Iran Registry of Clinical Trials (IRCT201009224794N1). The cohort was recruited in Tehran, an iodine-sufficient region, from March 1989 to February 2011. All patients had a diagnosis of diffuse goiter and hyperthyroidism based on the symptoms and signs, elevated serum free thyroxine (fT4), and/or serum total triiodothyronine (T3), suppressed TSH, and increased serum TSH receptor antibody (TRAb) or even distribution of radionuclide in thyroid scan. The exclusion criteria were hepatic, renal, or cardiovascular disease, pregnancy, and breastfeeding. Two hundred and twenty patients were needed for a small to medium effect size of 0.40, with 80% power and 5% significance, with a 10% chance of attrition. Two hundred and eight patients completed the study. The power of the post hoc test for this number of patients with the same effect was calculated to be 81%. The medical records of 121 Graves’ patients from 1989 to 2017 in Azizi clinic in Tehran who had total thyroidectomy were reviewed in detail. Seventeen patients who were lost to follow-up or had incomplete medical records were excluded. Finally, 104 post-thyroidectomy patients with Graves’ hyperthyroidism entered the study. In addition, 105 age- and sex-matched individuals from 178 Graves’ patients with LT-MMI treatment in the TOHID cohort from the same clinic were selected for comparison.

### 3.1. Surgical Group

The indications for thyroidectomy were: Patient's preference, allergy or intolerance to ATDs, and large goiters of more than 70 g by palpation. All patients were treated with 20 - 30 mg of MMI daily and attained clinical and biochemical euthyroidism and received Lugol's solution for 2 weeks before surgery. Methimazole was stopped at the time of thyroidectomy. One day after total thyroidectomy, levothyroxine was started with a daily dose of 1.6 µg/kg. Serum TSH concentration was measured 8 weeks postoperatively, and the dose of levothyroxine was adjusted. Patients were followed every 2 - 3 months for the first year after surgery, and the levothyroxine dosage was adjusted according to serum TSH concentrations.

### 3.2. Long-Term Methimazole Group

 For the first months, patients were treated with methimazole 20 to 30 mg per day. The dose was tapered at each subsequent visit to attain normal serum fT4, T3, and TSH concentrations (18).

After the first year, patients in both groups were followed every six months until the end of the study. At each visit, complaints were reviewed, compliance was evaluated, changes in weight and interactions with other medications were sought, and following a physical examination, serum concentrations of fT4, T3, and TSH were measured.

### 3.3. Study Outcomes

The primary outcomes were time to euthyroidism and time remaining in euthyroidism during the length of the study. The key secondary outcomes were subclinical and overt hypothyroidism and subclinical and overt hyperthyroidism.

### 3.4. Laboratory Measurements

During 2001 - 2005, serum fT4 and T3 were measured by radioimmunoassay (DiaMetra, Milan, Italy); TSH was measured by immunoradiometric assay using Izotop kits (Budapest, Hungary) and TRAb by enzyme-linked immunoabsorbent assay (Bio Vendor Laboratory Medicine Inc, Czech Republic). From 2006, all hormones and TRAb were measured by electrochemiluminescence immunoassay (Roche Diagnostics GmbH, Mannheim, Germany). We used the cutoff point of 1.75 IU/L for Elecsys^®^ TRAb assay, which has been proposed for diagnosis of Graves’ disease. All interassay and intraassay coefficients of variation were < 6.1% and < 9.1%, respectively.

### 3.5. Definitions

Hyperthyroidism was defined by a TSH concentration of < 0.4 mIU/L (reference range, 0.4-5.06 mIU/L), and fT4 concentration of > 23 pmol/L (reference range, 9 - 23 pmol/L), and/or a T3 concentration of > 200 ng/dL (reference range, 75 - 200 ng/dL); subclinical hyperthyroidism was characterized by a TSH concentration of < 0.4 mIU/L and serum concentrations of fT4 and T3 within the reference ranges. Overt hypothyroidism was defined as fT4 concentration of < 9 pmol/L and a TSH concentration above 5.06 mIU/L. Subclinical hypothyroidism was characterized by a TSH concentration of > 5.06 mIU/L and normal serum concentrations of fT4 and T3. Short-term (conventional) ATD therapy is defined as ATD therapy for 12-24 months, and LT-ATD (LT-MMI) treatment refers to treatment for ≥ 5 years.

### 3.6. Statistical Analyses

Characteristics of patients are presented as mean ± SD or frequency (%). Significant differences between the two study groups were assessed using analysis of variance and *t*-test for continuous variables and Mann-Whitney U, chi-square, and Fisher exact tests for categorical variables. The time to normalize serum concentrations of fT4, T3, and TSH was compared between the two groups to show the velocity of normalization. In addition, the percentage of time serum TSH remained within the reference ranges during > 11 years of follow-up was compared between the two groups. The time to euthyroidism in both groups was calculated from the start of MMI therapy until normal serum concentrations of TSH were achieved. The event date for thyroid status cases was described as the mid-time between the follow-up visit date at which particular thyroid status was detected and the most recent follow-up visit preceding the diagnosis. All analyses were performed using SPSS for Windows, version 19, with a 2-tailed P-value of < 0.05 considered significant.

## 4. Results

One hundred and four patients in the thyroidectomy group (80 women and 24 men) aged 39.12 ± 11.40 years, and 104 patients in the long-term methimazole (LT-MMI) group (82 women and 22 men) aged 39.11 ± 9.94 years were enrolled in this study. Table 1 shows the baseline characteristics of both groups. There was no statistically significant difference in age, sex, body mass index, smoking habit, ophthalmopathy, goiter size, serum concentrations of free thyroxine (fT4), triiodothyronine (T3), thyrotropin (TSH), and TSH receptor antibody (TRAb), and duration of follow-up between thyroidectomy and LT-MMI groups.

**Table 1.** Baseline Characteristics of Patients in the Thyroidectomy and Long-Term Methimazole Groups (N = 104) ^a^

Variables	Thyroidectomy	Long-Term Methimazole
**Age, y**	39.12 ± 11.40	39.11 ± 9.94
**Female**	84 (81)	84 (81)
**Body Mass Index (kg/m** ^**2**^ **)**	27.3 ± 5.3	27.5 ± 5.1
**Orbitopathy**	27 (26)	29 (28)
**Grade 2 Goiter**	63 (61)	61 (59)
**Current Smoking**	14 (13)	15 (14)
**fT4, pmol/L**	37.6 ± 8.8	38.2 ± 8.1
**T3, ng/dL**	390 ± 128	393 ± 124
**TSH, mU/L**	0.04 ± 0.03	0.03 ± 0.02
**TRAb, IU/L**	14.9 ± 8.3	15.2 ± 7.9
**Duration of follow up, months**	132 ± 39	131 ± 43

Abbreviations: fT4, free thyroxine; T3, triiodothyronine; TSH, thyrotropin; TRAb, TSH receptor antibody.

^a^ Values are presented as No. (%) or mean ± SD.

At the start of the study, patients were treated with a mean daily dose of 21 ± 1.1 mg methimazole (MMI); the mean dose was decreased to 5.1 ± 0.9, 3.9 ± 1.1, and 3.3 ± 0.8 mg by 2, 5, and 10 years of MMI treatment, respectively. The mean concentrations of fT4, T3, TSH, and TRAb at the end of the study were 16.1 ± 2.2 pmol/L, 119 ± 17 ng/dL, 2.5 ± 1.2 mU/L, and 1.1 ± 0.8 IU/mL, respectively.

### 4.1. Time to Euthyroidism

After the start of the intervention, the mean time to euthyroidism was 4.76 ± 2.68 months (range 2-16 months) in the thyroidectomy patients and 4.74 ± 2.42 months (range 2-16 months) in the LT-MMI patients (P-value: 0.92). It should be considered that all patients in both groups received MMI therapy to attain euthyroidism.

### 4.2. Time Remained in Euthyroidism

**Figure 1.**
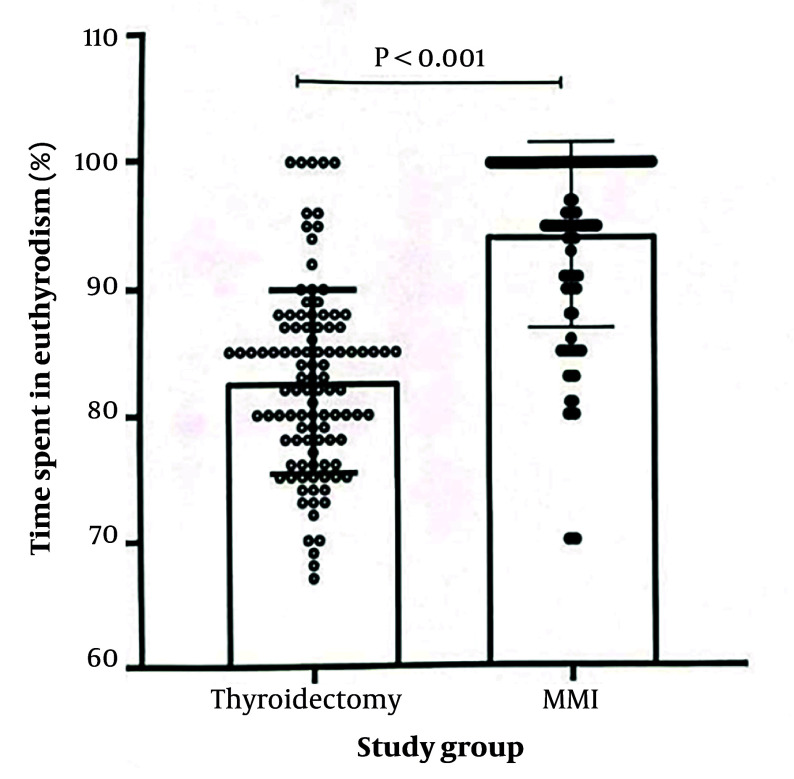
Individual values of time spent in euthyroidism during 11 years of follow-up in patients of thyroidectomy and long-term methimazole groups

Figure 1 shows that patients in the thyroidectomy group spent 82.67 ± 7.33% and those in the LT-MMI group spent 94.29 ± 7.33% of the time in euthyroidism during the study follow-up (P < 0.001). 

**Figure 2.**
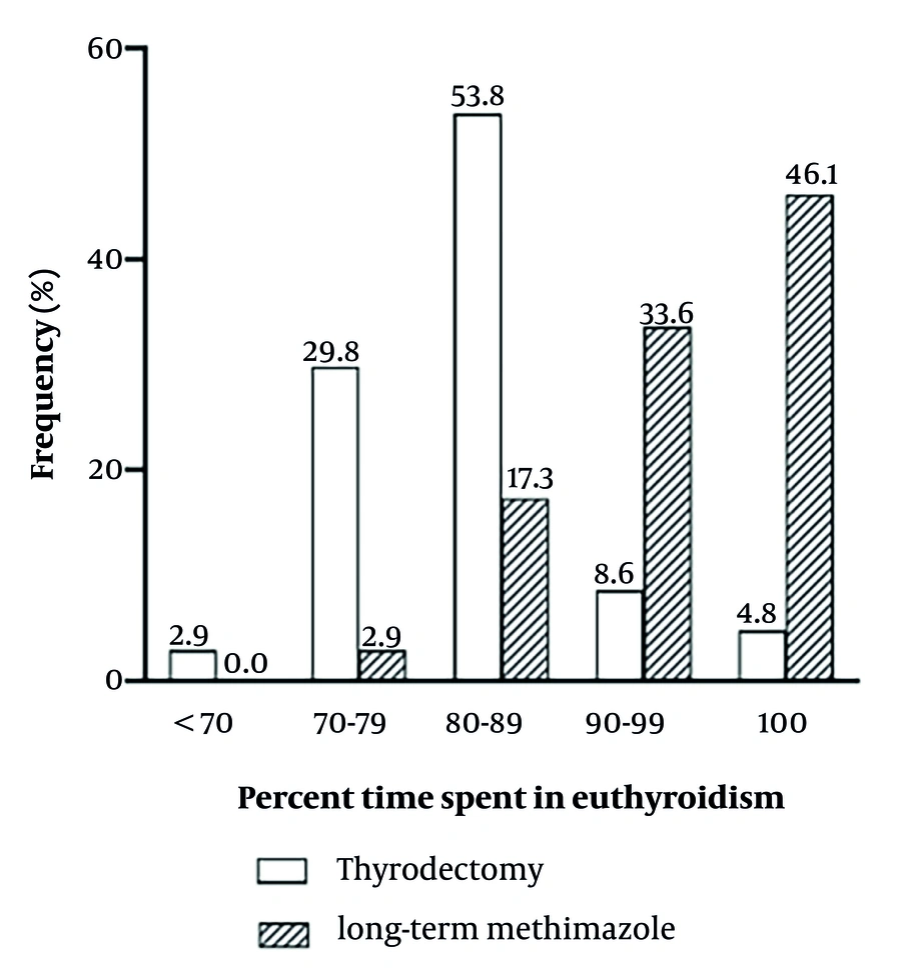
Frequency of percent time in euthyroidism in two groups of patients with Graves&rsquo; disease after total thyroidectomy and LT-MMI treatment.

The percentage of the time with TSH < 0.4 mU/L was 6.92 ± 6.7% and 5.47 ± 7.46% in the thyroidectomy and LT-MMI groups, respectively (P < 0.001). In addition, the percentage of time spent in TSH > 5 mU/L was also greater in thyroidectomy patients, as compared to LT-MMI patients: 3.99 ± 5.31% vs 0.27 ± 0.99% (P < 0.001), respectively (Table 2). While 80% of patients in the LT-MMI group spent ≥ 90% of the time in the euthyroid state, 87% of patients in the thyroidectomy group spent ≤ 89% of time in euthyroidism (Figure 2). 

**Table 2.** Percent of Time Spent in Various States of Thyroid Function in the Thyroidectomy and Long-Term Methimazole Groups

Variables	Long-Term Methimazole (n = 104)		Thyroidectomy (n=104)		P-Value
	Mean ± SD	Range	Mean ± SD	Range	
**Time to euthyroidism (months)**	4.48 ± 2.54	2 - 16	4.74 ± 2.42	2 - 12	0.92
**Time spent in euthyroidism (%)**	94.21 ± 6.98	70 - 100	82.67 ± 7.33	67 - 100	< 0.001
**TSH < 0.4 mIU/L (%)**	5.47 ± 7.46	0 - 30	6.92 ± 6.70	0 - 26	< 0.001
**TSH > 5.0 mIU/L (%)**	0.27 ± 0.99	0 - 5	3.99 ± 5.31	0 - 22	< 0.001
**Overt hypothyroidism (%)**	0.0 ± 0.0	0 - 0	2.87 ± 3.21	3 - 10	< 0.001
**Overt hyperthyroidism (%)**	0.0 ± 0.0	0 - 0	3.52 ± 4.67	5 - 15	< 0.001

**Figure 3.**
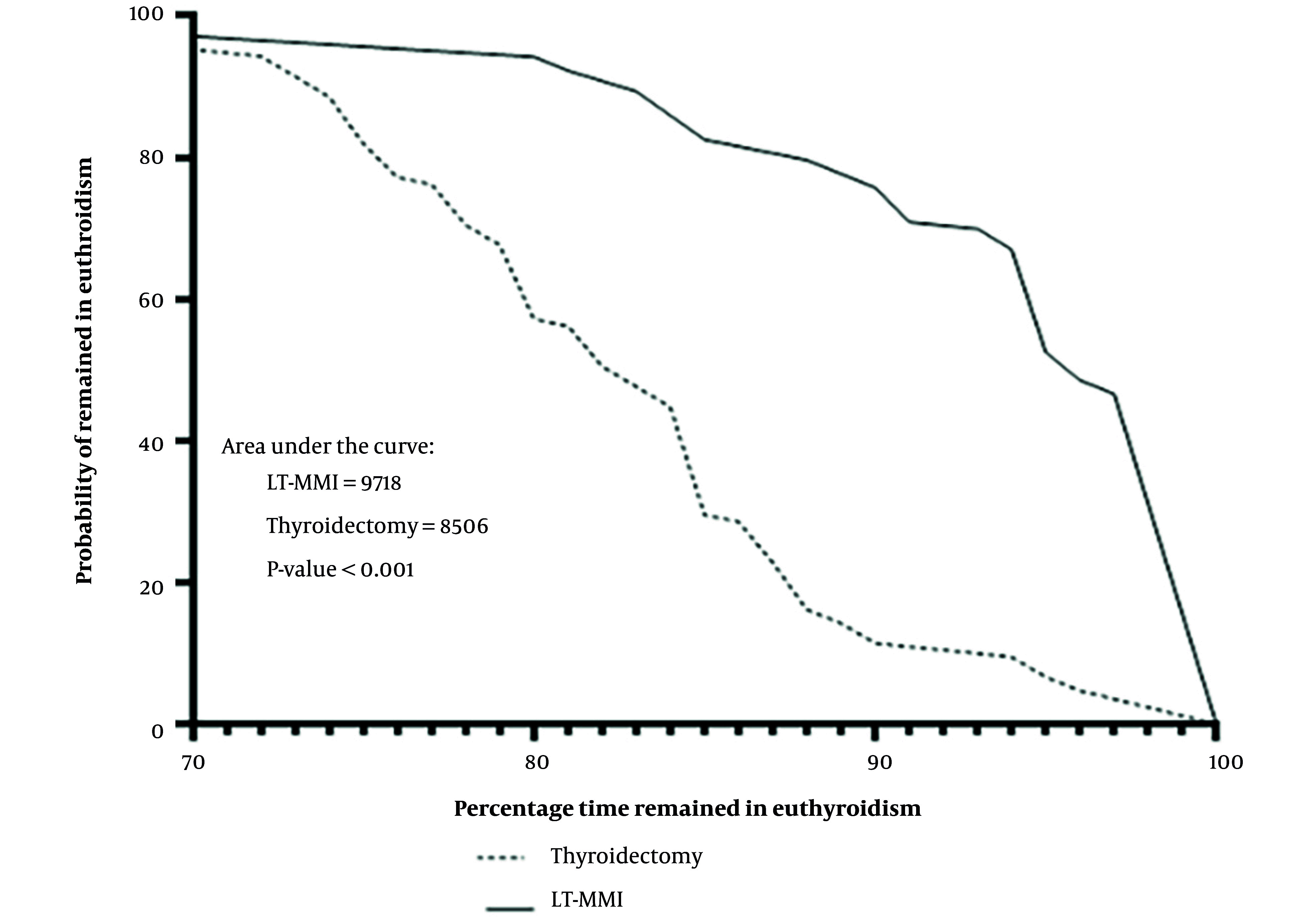
Comparison of the area under the curve (AUC) for time spent in euthyroidism between the two study groups

Figure 3 illustrates the area under the curve (AUC) for time spent in euthyroidism between the two study groups. Data showed that the start of levothyroxine (LT4) treatment after total thyroidectomy had a minor and non-significant effect on the time spent in euthyroidism, because all patients were euthyroid before surgery and an adequate dose of LT4 was started one day after thyroidectomy. Time spent in euthyroidism had no association with change in weight and addition of other medications to the management of patients.

### 4.3. Adverse Events

Among patients in the thyroidectomy group, during MMI treatment prior to surgery, four patients developed minor adverse events in the first 3 months of treatment. Ten patients in the thyroidectomy group experienced transient hypocalcemia, and one patient was re-operated because of hematoma. Permanent hypocalcemia and vocal cord paralysis occurred in 2 and 1 patient, respectively. In the LT-MMI group, five patients experienced adverse effects deemed to be drug-related, all within the first 3 months of treatment; skin reactions developed in 4 patients and were effectively relieved with antihistamines, and one had transiently elevated liver enzymes. No side effects were observed from month 4 through the end of 120 months on MMI. No serious complications such as agranulocytosis, hepatotoxicity, or vasculitis were observed.

### 4.4. End of Study

After the intervention, subsequent worsening or new onset of thyroid eye disease was 4 cases in each group. The mean change in body weight at the end of follow-up was 1.3 ± 3.2 and 0.49 ± 3.41 kg in the thyroidectomy and LT-MMI groups, respectively (P-value was not significant).

## 5. Discussion

The present study is the first report in the literature regarding the comparison of time to euthyroidism and time remaining in euthyroidism in patients with Graves’ disease managed by thyroidectomy with those treated with long-term methimazole (LT-MMI). The results showed that although there is no difference in the time to euthyroidism between the two groups, the percentage of time remaining in euthyroidism is significantly longer in patients in the LT-MMI group compared to those in the thyroidectomy group during 11 years of follow-up. Patients who underwent thyroid surgery experienced more time in both subclinical and overt hypothyroidism than those in the LT-MMI therapy. In addition, infrequent but important adverse events such as hypoparathyroidism and vocal cord paralysis occurred in patients treated with thyroidectomy.

Many elegant studies have concluded that cardiovascular safety should be an essential aspect of managing hyperthyroidism (10-14). It has been reported that all-cause mortality is increased in hyperthyroid patients affected by both diffuse toxic goiter and toxic multinodular goiter (12). Increased risk of mortality and cardiovascular morbidity such as heart failure, cardiac arrhythmias, and stroke have been documented in patients with uncorrected hyperthyroidism (3). All-cause mortality was increased in patients treated with radioactive iodine (RAI) or conventional 12 - 24 months of antithyroid drug (ATD) (11). Longer duration of suppressed serum thyrotropin (TSH) concentration may be associated with increased cardiovascular outcomes in both groups of treated and untreated patients with hyperthyroidism (10, 13). Interestingly, it has also been shown that early and effective control of hyperthyroidism is associated with improved survival, irrespective of the mode of treatment (14). Achieving a rapid euthyroid state and continuing with sustained euthyroidism in the follow-up should be planned and implemented during the care of patients with hyperthyroidism (13, 14).

Two recent studies have compared outcomes of patients with Graves' disease following ATD, RAI, and thyroidectomy. Liu et al. conducted a population-based retrospective analysis of patients with Graves’ disease in Hong Kong hospitals between 2006 and 2018. Over a median follow-up of 90 months with 47,470 person-years, the records of 6385 Graves’ patients who received first-line treatment of conventional ATD (57%), RAI (20%), and thyroidectomy (5%) were analyzed; patients who received thyroidectomy had lower chances of all-cause mortality, cardiovascular disease, atrial fibrillation, diabetes, hypertension, and psychological disease, as compared to ATD or RAI therapy. In addition, the relapse rate and health care cost were lower in the surgical group than the other groups (15). The limitations of this study were insufficient data sources such as Body Mass Index (BMI), serum thyroxine (T4), triiodothyronine (T3) concentrations, and smoking; inherited biases due to the retrospective nature of the study; and the limitation of generalizability of direct health care costs. In addition, the patients in the ATD group had been treated with the conventional ATD regimen. Another study was reported by Peng et al. using the Taiwan National Health Insurance research database on Graves’ patients between 2011 and 2020. Among 114,062 patients with the diagnosis of hyperthyroidism, 93.9% received ATD alone and 1.1% and 5.1% underwent RAI and thyroidectomy, respectively. Patients treated by surgery had a significantly lower risk of major adverse cardiovascular events (MACE), all-cause mortality, heart failure, and cardiovascular mortality, compared with patients treated with ATD. In addition, RAI treatment was associated with lower MACE risk than ATD therapy (16). Limitations of this study were reliance on intervention classification of diseases (ICD) codes for diagnosis of hyperthyroidism; retrospective nature of database study and limited access to detailed patient medical records; lack of availability of important variables such as BMI, smoking status, and lifestyle; uncertainty regarding the choice of treatment modality; small number of patients in the RAI group because of infrequent use of RAI in Taiwan; and lastly, treatment with conventional ATD rather than long-term ATD (LT-ATD) therapy.

We have previously compared the status of sustained euthyroidism during ATD treatment in the short-term (conventional) and LT-ATD therapy; 128 patients received 19 months and 130 patients were treated with 36 - 102 months of methimazole (MMI) and followed for 132 months. The time spent in euthyroidism was 90.4 ± 8.1% in the conventional group and 95.8 ± 7.0% in the LT-MMI group (23). It was also reported (23) that the time remaining in euthyroidism is much longer in those treated with LT-ATD (54.5 ± 7.3%) as compared to Graves’ patients treated with RAI (82.5 ± 11.0%, P < 0.001). In the present study, the time spent in euthyroidism was greater in those treated with LT-ATD (94.29 ± 7.37%) when compared to patients treated with thyroidectomy (82.67 ± 7.33%, P < 0.001). It is noteworthy that the time to reach euthyroidism after the start of treatment modality is not significantly different in patients treated with conventional, LT-ATD, or thyroidectomy patients who received ATD therapy before surgery; however, the time to euthyroidism is much longer in patients treated with RAI as compared to those treated with LT-ATD (3, 19). Table 3 shows a comparison of time to euthyroidism and time remaining in euthyroidism in four treatment modalities. The superiority of LT-ATD over the other three regimens is that the time remained in normal thyroid status (sustained euthyroidism) is much longer in the LT-ATD therapy compared to other treatment modalities in patients with Graves’ hyperthyroidism.

**Table 3.** Percent of Time Spent in Various States of Thyroid Function After Treatment of Graves’ Hyperthyroidism with Radioiodine, Thyroidectomy, Conventional (18 - 24 Months) and Long-Term (≥ 60 Months) ATD Treatment, During More Than 11 Years of Follow-up

Treatment of Hyperthyroidism	Reference No.	Percent Spent				
		Euthyroidism	TSH < 0.4 mU/L	TSH > 5.0 mU/L	Overthypothyroidism	Overthyperthyroidism
**Radioiodine**	(19)	82.5	9.1	7.3	0.7	0.4
**Thyroidectomy**	Present study	82.3	6.9	4.0	2.9	3.5
**ATD (conventional)**	(24)	90.4	6.7	2.9	0	0
**ATD (long-term)**	Present study	94.2	5.5	0.3	0	0

Abbreviation: TSH, thyrotropin.

There is a lack of trials comparing outcomes including quality of life, all-cause and cardiovascular mortality, and major cardiovascular events between the LT-ATD and RAI or surgical treatment modalities in patients with hyperthyroidism; however, with longer periods of sustained euthyroidism in the LT-MMI, it may be assumed that outcomes of this mode of treatment would be more favorable than RAI or surgical ablation. In addition to cardiovascular safety, adverse events and morbidities associated with each treatment modality should be considered. Major adverse events (agranulocytosis, hepatotoxicity, vasculitis, or pancreatitis) and minor complications (rash, arthralgia, and gastric intolerance) occur mainly in the first 3-4 months after the beginning of ATD treatment. It has been shown that in 1660 patients of 12 studies on LT-ATD treatment, 123 and 13 minor and major adverse events occurred in the first year, respectively; however, only 4 minor and one major (due to propylthiouracil) events occurred after one year until a mean of 5.8 years of LT-ATD therapy (25).

It has been reported that approximately 40% of hypothyroid patients on levothyroxine may not be in a euthyroid state (24). Increased body weight, decreased resting energy expenditure, impaired psychological well-being, and increased serum T4 to T3 ratio and rate of dyslipidemia occur in those with radioiodine-induced hypothyroidism, as compared to control healthy subjects or patients with euthyroid Graves’ disease (26-30). Thyroidectomy may induce immediate or long-term complications; 9.6 - 19.4% transient hypocalcemia and 0.9 - 1.4% permanent hypocalcemia due to hypoparathyroidism and 1.1-6.9% transient vocal cord paralysis, and 0.7 - 1.4% permanent recurrent nerve paralysis are the major complications of thyroid surgery. In addition, reoperation due to hematoma and occasional tracheostomy may occur (31, 32). Following recommendations of total thyroidectomy for patients with hyperthyroidism, almost all patients change to hypothyroid state and adverse events related to levothyroxine treatment occur following total thyroidectomy, similar to post-RAI hypothyroid treatment (26-30).

The strength of this study is that a knowledge gap in terms of cardiovascular events after various treatment modalities for hyperthyroidism exists, and we aimed and executed this study to address the question “Is early ablation therapy for Graves’ hyperthyroidism superior to ATD therapy?” The finding of longer periods of sustained euthyroidism induced by LT-ATD compared to ablation therapy (RAI and thyroidectomy) may be helpful in the decision-making of patients and physicians to adopt the proper mode of therapy for Graves’ hyperthyroidism.

This study has some limitations. First, the study was not double-blinded and the possibility of selection and assignment biases may exist. Second, due to limitations in the number of visits and frequency of laboratory assessments, the exact time of euthyroidism may be different from that found in this study. Third, the percentages of adverse events may not be accurate because of the limited number of patients in both study groups. Fourth, because of the low number of participants, we were not able to obtain appropriate data for outcomes related to mortality and MACE. Fifth, many cases included in this study are mainly non-severe cases of disease and the results may not be extended to severe Graves’ patients. Sixth, single-center results may carry potential bias. Finally, many patients did not have regular TRAb measurements; therefore, the course of decline in TRAb could not be compared.

We conclude that in patients with Graves’ hyperthyroidism, LT-ATD therapy is associated with more sustained normal TSH levels during 11 years of follow-up compared with total thyroidectomy.

## Data Availability

The dataset presented in the study is available on request from the corresponding author during submission or after publication.

## Clinical Trial Registration Code:

IRCT201009224794N1.
